# Representation of probabilistic scientific knowledge

**DOI:** 10.1186/2041-1480-4-S1-S7

**Published:** 2013-04-15

**Authors:** Larisa N Soldatova, Andrey Rzhetsky, Kurt De Grave, Ross D King

**Affiliations:** 1 Department of Information Systems and Computing, Brunel University, London, UK; 2 Department of Medicine & Department of Human Genetics, the University of Chicago, USA; 3 Department of Computer Science, KU Leuven, Belgium; 4 Manchester Institute of Biotechnology, the University of Manchester, UK

**Keywords:** ontology, knowledge representation, probabilistic reasoning

## Abstract

The theory of probability is widely used in biomedical research for data analysis and modelling. In previous work the probabilities of the research hypotheses have been recorded as experimental metadata. The ontology HELO is designed to support probabilistic reasoning, and provides semantic descriptors for reporting on research that involves operations with probabilities. HELO explicitly links research statements such as hypotheses, models, laws, conclusions, etc. to the associated probabilities of these statements being true. HELO enables the explicit semantic representation and accurate recording of probabilities in hypotheses, as well as the inference methods used to generate and update those hypotheses. We demonstrate the utility of HELO on three worked examples: changes in the probability of the hypothesis that sirtuins regulate human life span; changes in the probability of hypotheses about gene functions in the *S. cerevisiae* aromatic amino acid pathway; and the use of active learning in drug design (quantitative structure activity relation learning), where a strategy for the selection of compounds with the highest probability of improving on the best known compound was used. HELO is open source and available at https://github.com/larisa-soldatova/HELO

## Introduction

“All knowledge resolves itself into probability”.

David Hume, in a treatise of Human Nature (1888), 181-182.

Scientific knowledge is inherently uncertain: experimental observations may be corrupted by noise, and no matter how many times a theory has been tested there is still the possibility that new experimental observations will refute it — as famously happened to Newtonian mechanics. Probability theory has from its conception been utilized to represent this uncertainty in scientific knowledge. However the role of probability theory has proved controversial, with for example the great philosopher of science Karl Popper arguing that probabilities cannot be applied to scientific theories on the grounds that an infinite number of theories can explain any scientific data, therefore their *a priori* probabilities are zero. This view is now generally disregarded and a Bayesian approach to the use of probabilities in science is widely accepted. In Bayesian reasoning *a priori* probability estimates for hypotheses are updated through observation of additional evidence [1]. The Bayesian approach is arguably the only rational method for updating beliefs [2,3].

Despite the undoubted importance of probabilities in science it is unfortunately the case that conventional knowledge representations in bio-medicine are insufficient to support probabilistic reasoning. The best available representation, in our view, is the Evidence Code Ontology (ECO) [4]. ECO enables the recording of evidence that supports scientific statements, e.g. experimental evidence, sequence similarity, curator inference; and also by what method the evidence was obtained, e.g. through computational combinatorial analysis, inference from background knowledge, non-traceable author statement. This information enables researchers to qualitatively evaluate the degree of uncertainty of scientific statements. However, such evaluations are rarely recorded, not checked for consistency with other relevant evaluations, and therefore are difficult to use for probabilistic reasoning. There is a need for a resource that would enable the explicit quantitative recording of probabilities associated with research statements. To address this need we propose the ontology HELO (HypothEsis and Law Ontology) that supports probabilistic reasoning about bio-medical research statements.

## HELO aims

The HELO ontology was originally designed to support development of Robot Scientists, these are physically implemented laboratory automation systems that exploit techniques from the field of artificial intelligence to execute cycles of scientific experimentation.

A probability that a research statement is true may vary greatly depending on the source of the statement. While experimental data from good laboratories are likely to be true, even research statements extracted from very high impact journals are not necessarily valid. C.G. Bengley and L.M. Ellis in their recent article in *Nature* report that scientific findings have been confirmed only in 6 out of 53 “landmark” studies in haematology and oncology [5]. This is consistent with results in other areas. For example Prinz *et. al* report that only 25% of published pre-clinical studies could be validated [6]. The authors stressed that validation attempts could fail for various reasons, including technical differences. HELO aims to provide a framework for the recording of probabilities that research statements are true, and for probabilistic reasoning with such statements.

## The key HELO classes

### Research statements

The HELO representation of research statements is based on the representation of research hypothesis as PREDICATE(*entity_i_*, *entity_j_*) defined in an ontology LABORS, where *predicate* is a relation and *entity* is a class or instance defined in a domain ontology [7]. HELO enables one to formulate complex research statements, where basic (atomic) statements like PREDICATE(*entity_i_*, *entity_j_*) are combined by logical operators ˄, ˅, ¬, →, ↔. Entities that form research statements may be replaced by more generic entities (parent classes) and/or be specialized by their properties: individual gene names could be replaced with classes from Gene Ontology (GO) [8]; specific environmental factors could be replaced with general terms such as increased/decreased temperature, carbon source, addition of drugs, etc.; and measurable phenotypes could be replaced with general terms such as relate to growth, cell shape, etc. The following complex statement about yeast strains could be then represented: *“If all genes with lactase activity are deleted from a yeast strain and if this strain is grown in medium with lactose as the sole carbon source*, *then the phenotype will be no growth.”* This could be expressed in logic using terms defined in various ontologies as:

In combination with a logical model of metabolism these statements would enable deduction of the fact:

HELO defines a hierarchy of research statements: *research hypothesis*, *hypotheses set* (a collection of hypotheses with a total probability 1, it usually combines research hypotheses, negative hypotheses, and alternative hypotheses, see [7] for more detail), *assumption*, *conclusion*, *scientific law* (models and generic rules, including Bayes rule), *theorem* (including Bayes theorem). Research laws may be represented as production rules (*statement_i_* → *statement_j_*), where statements correspond to hypotheses, evidence, conclusions. For example,

Research laws may be models that are produced for example by the Eureka system that outputs laws of nature [9].

HELO is designed to consistently accommodate scientific hypotheses and laws collected from different sources: interviews with scientists, web pages, research papers, databases, program codes. Any research statement in HELO has an associated probability of being true.

### Probability

Probabilistic reasoning is essential in biomedicine, e.g. the Ontology of Adverse Events (OAE) models *causal adverse event probability* (an information content entity that represents a probability that an adverse event is caused (induced) by a medical intervention) [10], the Mass Spectrometry (MS) structured controlled vocabulary developed by the HUPO Proteomics Standards Initiative models *modification probability* (a priori probability of a modification) [11]. However, the concept of a probability is not modeled consistently in biomedical ontologies. MS models the class *probability* as a subclass of the class *modification parameters*. The Parasite Experiment Ontology (PEO) defines the concept as a subclass of the class *statistical measure* and *data collection* (a statistical way of expressing knowledge or belief that an event will occur or has occurred) [12]. Computational Neuroscience Ontology (CNO) defines *probability* as the subclass of the class *model parameter*[13]. CNO has the class *mathematical concept* (“a thing that represents the different mathematical concepts used to represent models”), but for some reason probability is not considered as a mathematical concept. The Semanticscience Integrated Ontology (SIO) [14] defines the class *probability* as the subclass of the class *description*. SIO has the class *mathematical entity*, but again probability is not considered as such.

HELO follows the theory of probability [15], [16] and defines the class *probability* as a subclass of the class *mathematical function* to enable mathematical operations with probabilities (a mathematical function expressing knowledge or belief that a research statement is true). This definition covers frequentist probabilities (taken as a limiting frequency of experimental observations), and also Bayesian “subjective” probabilities, or beliefs.

Reliable statistical estimates of the probability of a statement being true are often unavailable. In the subjective Bayesian framework human experts are expected to provide priors that capture scientific background knowledge and intuition. Obtaining such probabilities is notoriously difficult and there is an extensive literature on the subject. Once prior probabilities are given the probabilities of scientific statements may be then iteratively updated (increased or decreased) with new evidence. It is important to record these changes in value and how they were inferred.

HELO enables the recording of how probabilities were obtained. The class *method of probability estimation* has subclasses *Bayesian inference*, *expert estimation*, *statistical calculation*, *deduction*, *abduction*, *induction*, *homological inference;* and linked to the class *procedure* that records a specific algorithm implementation for obtaining a probability of a research statement. The class *probability* has the subclasses *prior probability* and *posterior probability*. A *research statement* is linked to an associated *probability* via the functional relation HAS-PROBABILTY.

**Figure 1:**
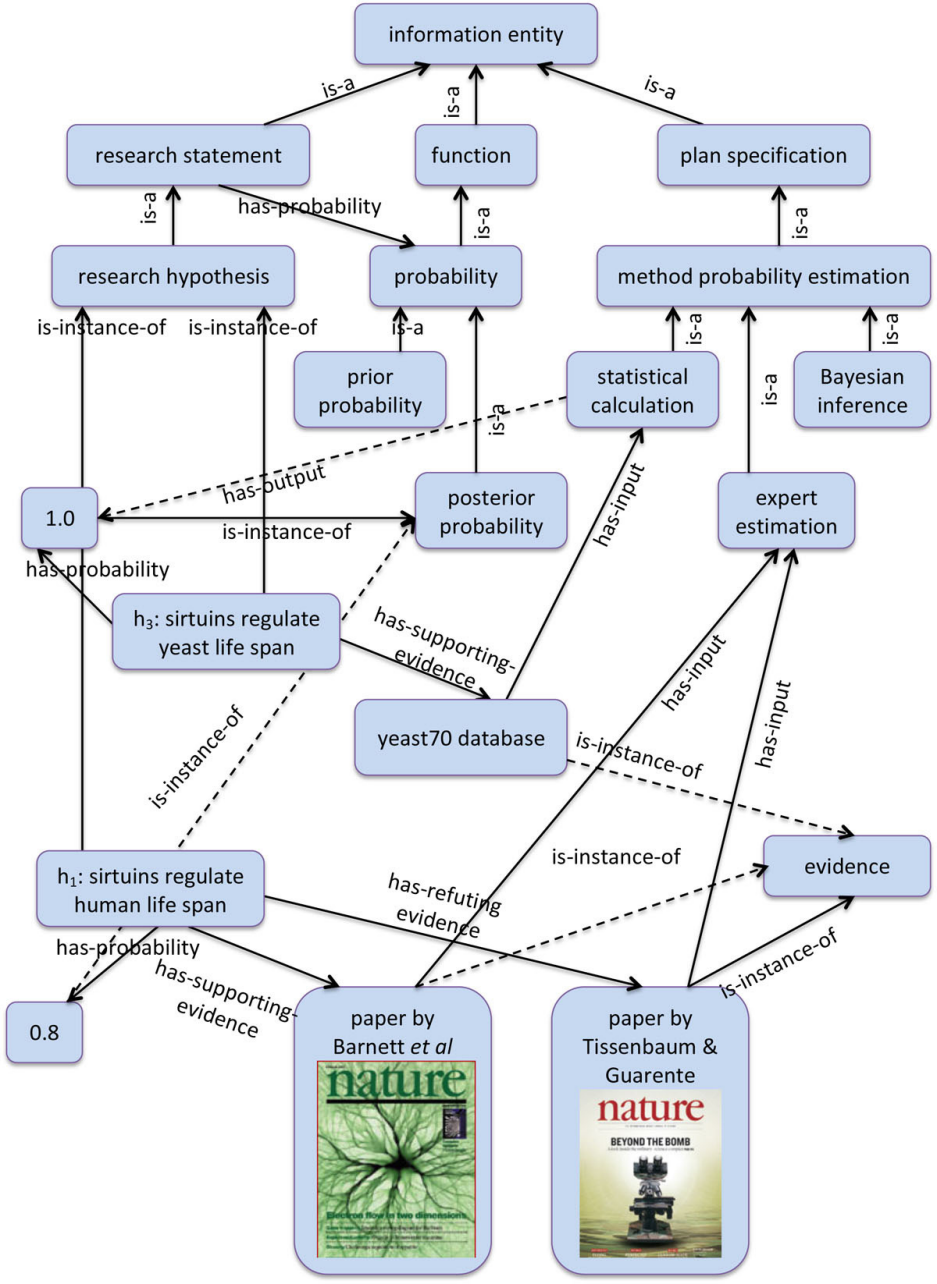
**An example of the HELO representation of a research statement**. The figure shows the representation of the values of the prior and posterior probabilities of the research statement about sirtuins, and also the supporting and refuting evidence.

HELO imports from SIO the relations refutes, supports, disputes to link research statements, and the relations HAS-DISPUTING-EVIDENCE, HAS-REFUTING-EVIDENCE, HAS-SUPPORTING-EVIDENCE to link research statements and evidence (see Fig.1).

### An ontology of the theory of probability

**Figure 2:**
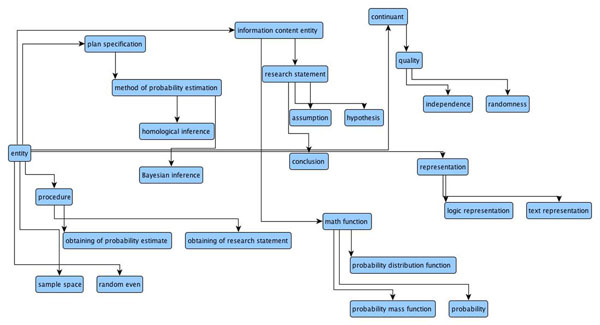
**An overview of the ontology HELO**. The figure shows the top-level classes of HELO and some of their extentions.

HELO defines the key entities of the theory of probability to enable logically consistent recording of operations that involve probabilities. HELO includes such classes as *variable*, *probability distribution function*, *probability mass function*, *mean*, *variance*, and such qualities as *independent*, *random* (*variable*), *joint* (*probability*). In order to organize these classes into a hierarchical system, HELO imports the following top-level classes: *continuant* (from BFO [17]), *information content entity* (from IAO [18]), *plan specification* (from OBI [19]), *procedure* (from LABORS [20]), *representation* (from LABORS and SIO [14]) (see Fig.2).

Additionally, the class *random event* is defined as an upper class, because the concept of an *event* ontologically differs from the notion of a *process* or any other notion. It may involve a process and participants and it has an associated time point, e.g. the end of the process. The theory of probability deals with *random events* defined on a *sample space* of all possible outcomes of a random event.

HELO is expressed in OWL-DL. It has been checked for logical consistency with the reasoners HermiT 1.3.6 and FaCT^++^. HELO is open source and available at https://github.com/larisa-soldatova/HELO.

## Worked examples

### The S. cerevisiae aromatic amino acid pathway

This example demonstrates how a probability that a research hypothesis is true is used for automated experimentation.

King *et al*. (2009) demonstrated the full automation of scientific discovery [20]. The Robot Scientist “Adam” employed abductive inference to formulate a set of 8 hypotheses based on its logical model of the *S*. cerevisiae aromatic amino acid (AAA) pathway concerning which gene had been deleted (see Supplementary Information in [20] for more detail). The *prior probability* of each hypothesis from the set of being correct, (using a uniform distribution) was 1/8. Adam then planned and executed cycles of auxotrophic experiments to test these hypotheses. Each cycle resulted in the rejection of one or more hypotheses, and the probabilities of the remaining hypotheses were increased with each cycle. The experiments were executed until only one hypothesis was left. The *posterior probability* of the remaining hypothesis was 1 and all of the others - 0.

In making its decision about which experiment to execute in each cycle Adam used the probabilities of the hypotheses being true, the cost of the compounds required in the experiments to test those hypotheses, and the predicted information gain in testing the hypotheses. Previously, probabilities of research hypotheses were represented and recorded as associated with the experiment’s metadata [21]. HELO enables the direct recording of *prior* and *posterior probabilities* as properties of research hypotheses. This makes the representation of probabilistic knowledge explicit, and streamlines probabilistic reasoning, decision making, and automated experimentation.

### Sirtuins

We use the example of sirtuins as an example of how to utilise HELO for probabilistic representation of research statements. We are interested in recording and automating the argumentation involved in the sirtuin case, both to direct our own research into aging, but also as an exemplar of biological reasoning. This example is typical in how the probability of scientific statements varies over time with the observation of new experiments. The example also illustrates the use of homologous inference, which is the basis of much biological reasoning, and which is essentially probabilistic.

Sirtuins are highly conserved *NAD*^+^ - dependent deacetylases that are believed to play a role in regulating lifespan in many organisms. The potential role of sirtuins in extending human lifespan has led to extensive research into the human gene SIRT1 and its orthologs. For example in 2001 Tissenbaum & Guarente showed that increased dosage of the SIRT1 homolog extends lifespan in the nematode (*Caenorhabditis elegans*) [22]. Increasing sirtuin level through genetic manipulation has been observed to extend lifespan in *C. elegans*, the yeast (*Saccharomyces cerevisiae*), the fruitfly (*Drosophila melanogaster*), and the mouse (*Mus musculus*) [23,24]. This research sparked commercial interest and in 2008 Sirtris Pharmaceuticals Inc., working on exploiting sirtuin modulation for the treatment of human disease, was bought by GlaxoSmithKline for approximately USD 720 million.

This excitement about the potential of sirtuins suffered a major setback in 2011 when Burnett *et al*. reported that overexpressing the sirtuin gene in two model organisms, *C. elegans* and *D. melanogaster* did not in fact boost longevity as had been previously reported [25]. The situation changed again in 2012 when Kanfi *et al*. reported that the sirtuin SIRT6 regulates lifespan in male mice, but not in female ones [23]. Therefore the probability of the research hypothesis that sirtuins regulate organism lifespan has increased and decreased over the last decade.

The primary research hypothesis *h*_1_ we are interested in is: “SIRT1 regulates human life span” (SIRT1 is a sirtuin gene in humans). HELO enables the recording of this research statement:

However, it is difficult to directly test this hypothesis, so most evidence relating to it comes from laboratory experiments using model eukaryotes. For example a hypothesis *h*_2_ is about *C. elegans*:

and a hypothesis *h*_3_ is about *S. cereυisiae:*

The evidence about *h*_2_ and *h*_3_ is then related to *h*_1_ by probabilistic reasoning (homological inference):

SIR2 is the *S. cereυisiae* homolog of SIRT1. The research hypothesis *h*_3_ “SIR2 regulates yeast life span” is very well supported by the scientific literature. The dataset yeast70 is a subset of Gene Ways 7.0 database [26]. The Gene Ways 7.0 database was produced through automated analysis of 368,331 full-text research articles and 8,039,972 article abstracts from the PubMed database, using the GeneWays system. The database covers a wide spectrum of molecular interactions, such as bind, phosphorylate, glycosylate, and activate (nearly 500 relations in total). The dataset yeast70 has 1,135 sentences containing the keyword “aging” and yeast gene names, 492 of them contain SIR2, the gene SIR1 is not mentioned, and SIR3 is mentioned 42 times. Examining these papers suggests that the probability of *h*_2_ is close to 1.0.

The probability of scientific hypotheses changes with new evidence, and we wish to use HELO to represent this. For example Burnett *et al*. results directly decreased the probability of the hypotheses regarding the function of the SIRT1 homologs in *Caenorhabditis elegans* and *Drosophila melanogaster*, and these indirectly decreased the probability of *h*_1_. The situation changed again in 2012 with Kanfi *et al*. where the evidence directly increased the probability of the hypotheses regarding the function of the SIRT1 homolog in *Mus musculus*, and this indirectly increased the probability of *h*_1_ (see Fig.1).

Of course the weight of the evidence in these papers on *h*_1_ depends on a host of factors other than simply the model species involved: the amount and variety of evidence, its statistical confidence, the lab where the work was done, the publisher, etc. Taking all these into account an expert estimate of the probability that *h*_1_ is held after the publication of the paper [22] is 0.8 (see Fig.1). It should be noted that the exact probability of *h*_1_, say 0.8 or 0.82, is not that critical. What is important is the “ball-park” figure, and the direction of change with new evidence. Our idea is that addition of more and more evidence and inferences to the argument constrain the probabilities to reasonable numbers. It is our contention that all human scientists make such implicit inferences and much is to be gained by making them explicit. In addition these probability can be used for further automated inference and experimentation.

Experiments with a Sir2 deletant strain run within the Robot Scientists project showed no difference between the wild type, while yeast strains with *NAD*^+^ grew to a significantly higher biomass than the wild type. The experiments demonstrate that Sir2 functions differently from other *NAD*^+^ genes, and this indirectly supports the hypothesis *h*_1_. It is clear that further experimentation is required to accept or reject the hypothesis *h*_1_.

### Active learning for drug discovery

This example demonstrates the recording of probabilities in drug discovery experiments. The goal of these experiments was to find the best compound (with respect to a biomedical assay, e.g. for treating cancer) without having to test all the compounds against the assay. This involved learning quantitative structure activity relationships (QSARs). These are functions that take as input the structure of a compound and output an estimate of how well the compound will perform in a biomedical assay. The investigation was computational and used existing assay results.

The task of finding the best instance (e.g. compounds, parameters) as evaluated on an unknown target function (e.g. high biological activity, minimal costs) using limited resources (e.g. time) is important to many scientific disciplines. In drug discovery it is not sufficient to find just a single best compound or “lead” as several leads improve the chances of finding a compound that passes toxicology tests. The challenge therefore is to identify the *k* best performing instances (= compounds in this context) using as few experiments as possible. We refer to this task as *active k-optimization.*

We applied machine learning to solve the *active k-optimization* task and to propose the best candidates for screening [27]. We considered several selection strategies for the best instances: Cox and John’s lower confidence bound criterion [28] (we refer to it as the optimistic strategy), the most probable improvement (MPI) of the current solution strategy [29], the maximum expected improvement (MEI) strategy, and also the random choice (see [27] for more detail).

These strategies were evaluated on the US National Cancer Institute 60 anticancer drug screen (NCI60) dataset [30]. This repository contains measurements of the inhibitory power of tens of thousands of chemical compounds against 59 different cancer cell lines (one of the originally 60 cell lines was evicted because it was essentially a replicate of another one [31]). NCI reports the negative log-concentration required for 50% cancer cell growth inhibition (pGI_50_) as well as cytostatic and cytotoxic effect measures, but we only used the pGI_50_.

The goal is to find compounds in a library that have a high pGI_50_, and to do so using as few pGI_50_ measurements as possible. The program bootstraps by selecting 10 random compounds and measuring their pGI_50_. In each subsequent step, a current QSAR model is fitted to all available pGI_50_ values. The model is used to predict the pGI_50_ for all remaining (untested) compounds in the library. The model is a Gaussian process, which outputs a (Normal) distribution for the pGI_50_ value rather than only a point prediction. This enables the implementation of the previously listed strategies. For example, for the MPI strategy, one computes the probability that a compound has a pGI_50_ which is larger than the current *k*-th best one. The compound with the highest probability is selected for the next measurement of pGI_50_.

**Figure 3:**
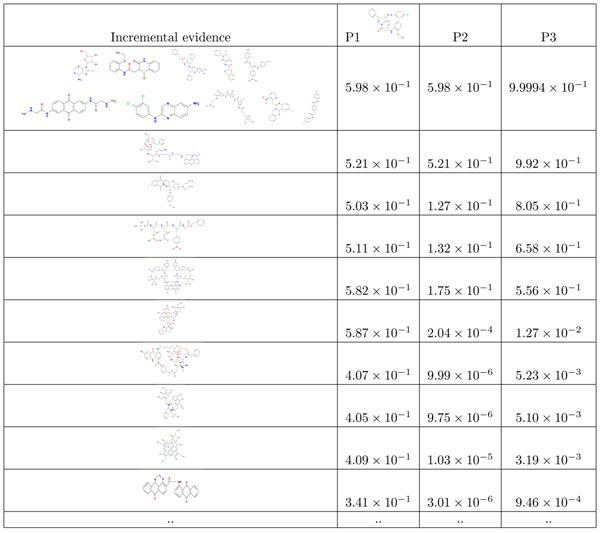
The probabilities that the selected compounds have high *GI*_50_

The table in Figure 3 illustrates MPI for a particular cell line 786-0, for a specific bootstrap, and for *k* = 1. The first column of the table shows the number of known pGI_50_ values at that time. *P*1 is the probability, given the current evidence, that a particular compound NSC 642567 will have a pGI_50_ better than the best bootstrap compound. The subsequent column shows what is the probability *P*2 that NSC 642567 has a better pGI_50_ than the current best value. The third column shows the highest such probability *P*3 for any of the compounds remaining in the library.

Each computational experiment was repeated 20 times and the results were averaged. Overall, on the NCI60 datasets, the optimistic strategy was most robust. In all situations considered, it performed either best or not significantly worse than the best strategy (see [27] for detail and diagrams). The performance of MPI is competitive for medium experimental budgets, but it may fail to find more than one good compound when constrained to low budgets, and it does not optimally exploit high budgets. MEI is a very good strategy when about 10 compounds are needed. The random selection strategy performs worse than all other selection methods in all settings. Actively choosing compounds substantially speeds up the finding of the compounds with high pGI_50_.

HELO enables the recording of these important results in a semantically defined way. The following semantic descriptors are required for the reporting of this study: Gaussian distribution, zero mean, variance, prior belief, posterior probability, random variable, likelihood, estimated probability. HELO contains exact matching terms or equivalent synonyms of the required semantic descriptors, for example HAS-VALUE(mean, 0) is equivalent to zero mean.

## Conclusion

Scientific knowledge is inherently uncertain. There is therefore a need for a representation that focuses on the probabilistic features of research statements, and supports probabilistic reasoning. In order to address this need we proposed the ontology HELO that supports probabilistic reasoning over uncertain scientific statements. HELO defines a hierarchy of typical research statements and links them to their associated probabilities, and methods of obtaining those probabilities. We demonstrated HELO on the representation of scientific belief that sirtuins regulate organism life span, and regarding deleted genes in the *S. cereυisiae* aromatic amino acid pathway. In both cases the probability of research statements changed with new evidence, and it is clearly important to employ the most updated probability estimate for making decisions about research involving these genes. The active learning for drug discovery study is based on operations with probabilities. The probabilities of having a high pGI_50_ were iteratively computed for all compounds in the library, and the best compounds were chosen for further study. HELO enables accurate recording of supporting and refuting evidence of research statements, and how they participate in the process of updating probability values.

HELO is specifically designed to support the cycles of automatic scientific discovery that incorporate text mining, machine learning, robotic automation, and knowledge representation, and may be of use for other of research that involve probabilistic reasoning.

## Author’s contributions

LNS originated the idea of ontological representation of the key entities of the theory of probability and worked on the ontology HELO. AR originated the idea of annotating research statements extracted from natural language text with semantic descriptors indicating the level of truthfulness of those statements, and also the recording of competing and contradictory statements. KDeG applied HELO for the reporting on the active learning for high throughput screening study. RDK originated the idea of using probabilities of hypotheses for the choice of experiments in automated experimentation. He also contributed to the development of HELO and other worked examples.

## Declarations

The publication costs for this article were funded by the corresponding author’s institution, Brunei University, London.

This article has been published as part of *Journal of Biomedical Semantics* Volume 4 Supplement 1, 2013: Proceedings of the Bio-Ontologies Special Interest Group 2012. The full contents of the supplement are available online at http://www.jbiomedsem.com/supplements/4/S1
